# Direct Tensile Behavior of Thermally Activated Concrete Reinforced with Short Iron-Based Shape Memory Alloy (Fe-SMA) Fibers: Experimental and Numerical Investigation

**DOI:** 10.3390/ma19214490

**Published:** 2026-10-25

**Authors:** Alireza Tabrizikahou

**Affiliations:** Institute of Building Engineering, Poznan University of Technology, Jacka Rychlewskiego 1, 61-131 Poznan, Poland; alireza.tabrizikahou@put.poznan.pl

**Keywords:** iron-based shape memory alloy (Fe-SMA), fiber-reinforced concrete, direct tensile behavior, thermal activation, internal prestressing, finite element modeling

## Abstract

Short iron-based shape memory alloy (Fe-SMA) fibers can act as active internal reinforcement after thermal activation. This study examined the direct tensile behavior of concrete containing 2.0% by volume of straight Fe-SMA fibers, geometrically identical steel fibers, or no fibers. ASTM C307 briquettes were tested under ambient conditions and after a 150 min hold at a chamber temperature of 160 °C, followed by cooling. Plain-concrete flexural and compressive tests characterized matrix degradation. Mean tensile strengths were 7.96 and 7.18 MPa for the unheated steel-fiber and Fe-SMA groups, respectively. After heating, these values were 5.89 and 9.41 MPa. The Fe-SMA increase was 31.1%, corresponding to a mean difference of 2.23 MPa with a 95% confidence interval of 1.66–2.80 MPa. A three-dimensional ABAQUS/CAE model with randomly distributed, fully bonded fibers reproduced the main response features using a recovery-stress target adopted from prior same-batch characterization. Separate numerical sweeps predicted higher strength with increasing fiber content and decreasing diameter within the investigated ranges. These results support the feasibility of thermally activated short Fe-SMA reinforcement. The numerical recommendations remain conditional on the idealized interface, adopted activation model, and investigated parameter ranges. Direct core-temperature monitoring and microscopic interface characterization remain necessary for further validation.

## 1. Introduction

Direct tensile behavior is one of the fundamental material responses governing crack initiation, crack bridging, post-cracking resistance, and constitutive modeling of fiber-reinforced cementitious composites [1,2]. Although flexural and splitting tests are widely used, direct-tension testing provides a more direct basis for identifying the uniaxial tensile response and tensile constitutive parameters. Studies of strain-hardening composites and UHPC have examined tensile stress-crack-opening behavior and constitutive modeling [3,4], while related FRCM research provides context for the mechanical characterization of cementitious composites [5]. The present study adopts the ASTM C307 briquette geometry [6] to investigate short Fe-SMA fibers under direct tension.

Activation gives SMA reinforcement a role beyond resisting an already open crack. A restrained shape memory contraction can introduce compression into the surrounding matrix, while suitable alloy systems can also support recentering and crack closure [7,8,9,10]. Whether these functions benefit a cementitious composite depends on the alloy, its processing history, the activation procedure, and the interface through which forces are transferred. Reviews identify these dependencies, together with fiber dosage and numerical representation, as unresolved practical issues [11,12].

Most earlier studies of SMA fibers in cementitious materials have focused on NiTi-based systems. Direct tensile studies have demonstrated the prestressing potential of short SMA fibers [13,14]. Related studies have investigated flexural capacity, crack closure, recentering, and pull-out behavior [15,16,17,18]. These studies establish a basis for active fiber reinforcement, although the cost of NiTi alloys can constrain broader civil-engineering applications.

The lower material cost of Fe-SMA relative to NiTi motivates its use in civil engineering [19,20,21]. Existing reinforcement applications include prestressed strips, shear-strengthening systems, near-surface-mounted reinforcement, and ribbed bars [22,23]. Processing Fe-SMA into thin wire extends this material family to dispersed short reinforcement [24].

Two earlier publications by the author provide the immediate background to this study. Tabrizikahou et al. [25] examined three-point bending of prisms reinforced with 35 mm hooked Fe-SMA fibers, including activation at 160 and 200 °C. That work also supplied the constrained-fiber characterization used here. Tabrizikahou and Białasik [26] subsequently investigated flexural reloading after pre-damage and thermal conditioning, including changes in crack width. Those studies addressed flexural prestressing and post-damage strengthening, respectively. Neither supplied the present comparison of initially undamaged ASTM C307 briquettes containing straight, geometrically matched Fe-SMA and steel fibers under direct tension.

A bending result does not directly determine the uniaxial tensile response because the stress distribution and crack development differ. Direct-tension evidence for SMA reinforcement exists, particularly for NiTi-based systems [13], but the response of short straight Fe-SMA reinforcement requires its own experimental assessment. Pull-out studies further show that changing the end geometry or thermal treatment can alter force transfer [27,28]. Consequently, the earlier hooked-fiber results cannot simply be assigned to 20 mm straight fibers. Here, matching length and diameter between the active and passive reinforcement removes nominal fiber geometry as a comparison variable; it does not imply identical interfaces or improved workability.

The original contribution is the direct-tension dataset and its interpretation for 20 mm long, 0.5 mm diameter straight fibers at 2.0% volume fraction, tested at ambient conditions and after a 160 °C chamber treatment followed by cooling. Plain-concrete and geometrically matched steel-fiber controls establish the corresponding strength changes without shape memory activation. Additional plain-concrete flexural and compression measurements characterize the matrix response. This design allows the net Fe-SMA tensile benefit to be assessed alongside thermal matrix degradation, although it does not directly measure the prestress in individual embedded fibers.

The study also applies a three-dimensional random fiber model to the briquette tests and examines fiber content and diameter in two separate numerical sweeps. Random fiber generation, concrete damage plasticity, and an equivalent contraction representation of activation are established modeling approaches; they are not claimed as new inventions here. The study-specific contribution is their application to the straight-fiber tensile configuration and the resulting conditional parameter trends. The Fe-SMA batch, processing information, and constrained recovery-stress basis are inherited from earlier work and are identified in Section 2.1 and Section 4.2.2. The present briquette measurements are distinct from the earlier flexural datasets. No new standalone fiber characterization or microscopic evidence is claimed.

## 2. Materials and Methods

### 2.1. Materials and Specimen Preparation

Each reinforced mixture contained a nominal fiber volume of 2.0%. Both fiber materials were supplied as straight segments, 20 mm in length and 0.5 mm in diameter (aspect ratio 40). These dimensions define the controlled geometric comparison; differences in stiffness, activation response, and interfacial behavior remain material-dependent.

Re-fer AG (Seewen, Switzerland) supplied the Fe-SMA from the batch characterized in [25]. Its nominal mass composition was Fe-17Mn-5Si-10Cr-4Ni-1(V,C). Processing comprised a 2 h anneal at 850 °C, following [24], and continuous wire prestraining to 5.0%±1.0%. The prestrained 0.5 mm wire was then cut to 20 mm without forming hooks. Thus, the shape memory prestrain preceded both cutting and concrete mixing. The passive steel fibers had the same dimensions; supplier information gave a tensile strength of approximately 1345 MPa and an elastic modulus of 210 GPa. Table 1 separates characterization reported elsewhere from the parameters assigned in the present simulations.

Table 2 specifies the concrete composition. A laboratory bowl mixer (produced by Matest S.p.A., Treviolo, Italy) operated at 25 rpm, with the mixing sequence based on ASTM C192 [29]. Once the matrix appeared homogeneous, fibers were fed in gradually to limit tangling during addition. A single batch was produced for each of the three reinforcement categories and divided between the ambient and heated groups. Molds were filled without vibration; no flow test or quantitative dispersion assessment was conducted. The first 24 h of curing took place in the molds at approximately 24 °C and 90% relative humidity. After demolding, specimens remained in water at 22–24 °C until 28 days after casting. These recorded conditions define the procedure used; they are not a claim of compliance with every curing provision of the standard.

ASTM C307 briquettes [6] were used for direct tension, and 40×40×160 mm prisms were used for flexure followed by compression of their halves according to EN 1015-11 [30]. Five briquettes were tested for each reinforcement–thermal condition combination. Five plain-concrete prisms were tested in each thermal condition, yielding ten compression tests on the resulting halves. Table 3 lists the groups. Replication was used to estimate within-batch scatter; it does not establish reproducibility across independent production batches. The mechanical test order was randomized.

### 2.2. Test Methods

**Figure 1:**
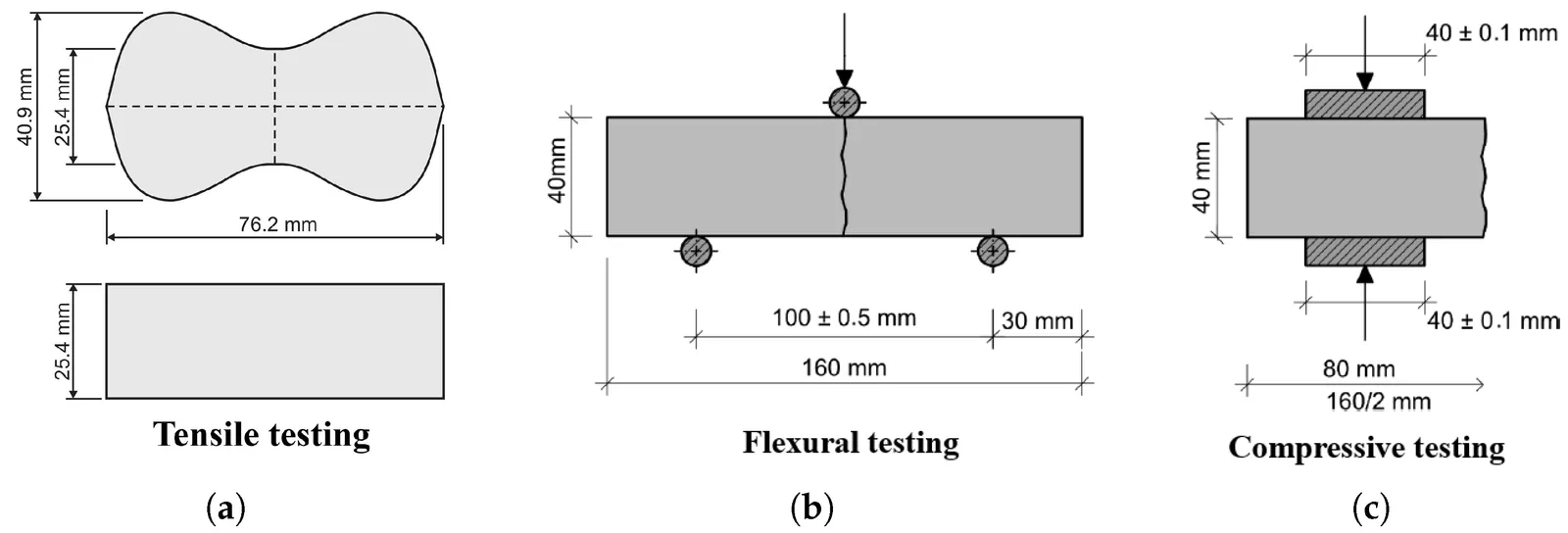
Specimen geometries and loading arrangements: (**a**) ASTM C307 briquette in tension; (**b**) EN 1015-11 prism in bending; (**c**) compression of a prism half. Only plain concrete was tested in (**b**,**c**).

Direct tension was applied to the ASTM C307 briquette geometry [6] shown in Figure 1a. This geometry was selected as a practical configuration for gripping the small specimens, recognizing that the standard concerns chemical-resistant mortars. The plain matrix was also tested in bending and compression using EN 1015-11 [30]: prisms measured 40×40×160 mm, and the halves remaining after flexure were loaded in compression (Figure 1b,c). These supplementary tests quantify the matrix response and provide the calibration data described in Section 4.

**Figure 2:**
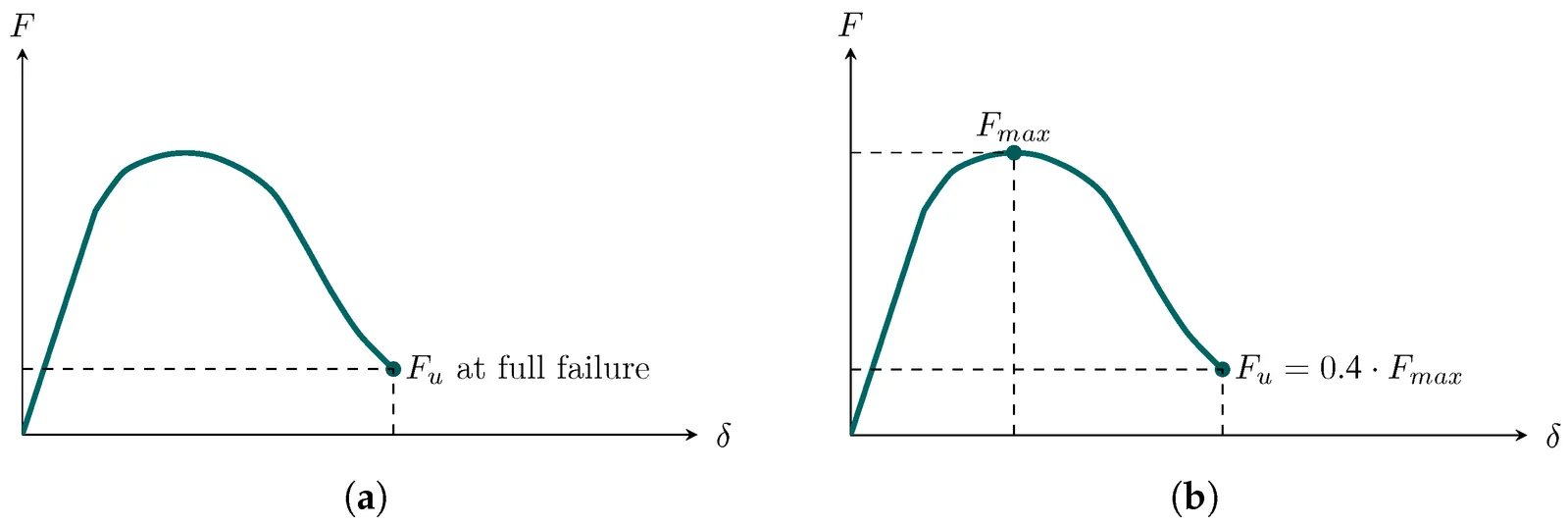
Loading and stopping conditions: (**a**) plain concrete taken to loss of capacity; (**b**) reinforced concrete stopped at 40% of peak load.

The actuator advanced at 0.5 mm/min in displacement control, and the machine logged force, actuator displacement, and time. This rate was used to follow damage development through and beyond the peak. Plain-concrete tests continued to separation and loss of load capacity (Figure 2a). For reinforced specimens, the machine stopped when the descending load reached 40% of the recorded maximum, equivalent to a 60% drop (Figure 2b). The reinforced and unreinforced tests therefore had different stopping conditions.

Following water curing, a 24 h laboratory conditioning period preceded heating; air conditions were 21–24 °C and 35–40% relative humidity. The specimens entered an unheated chamber on a rack with gaps for air circulation. A 150 min hold began only when the displayed chamber air temperature reached the 160 °C setpoint. Neither the preceding ramp nor temperatures inside the briquettes were logged. At the end of the hold, power was switched off and the specimens remained in the chamber during cooling. Removal took place approximately 4 h later, when the display indicated room-temperature air. Mechanical testing was deferred until the next day to allow further equilibration. The resulting strengths describe the cooled material, not its behavior while hot.

The duration was selected using earlier thermal measurements in the same chamber. Provided 40×40×160 mm prisms in [25,27] required approximately 150 min to approach the target core temperature. The present briquettes were smaller overall, and the additional 150 min hold after the chamber reached its setpoint was intended to provide sufficient equilibration time. These observations support the protocol but do not directly establish the present briquettes’ core-temperature history: the earlier mixtures were related cementitious systems, not identical mix designs, and geometry, moisture, and chamber loading also affect heat transfer. Related briquette pull-out experiments [31] used different activation temperatures and a 4 h hold, so they are methodological context rather than verification of the present 150 min hold. No claim of experimentally verified temperature uniformity is made here. Direct core monitoring in sacrificial briquettes is required in future work.

### 2.3. Statistical Analysis

The analysis uses one peak-strength value from each specimen. Group summaries comprise the arithmetic mean, the sample SD calculated with divisor n−1, the CV, and the smallest and largest observations. With five tensile or flexural specimens per group, the mean CI is $\bar{x}\pm t_{0.975,4}\;\mathrm{SD}/\sqrt{5}$ at the 95% level. Separate two-sided Welch tests compare the thermal conditions for plain-concrete tension, steel-fiber tension, Fe-SMA tension, and plain-concrete flexure. The four *p*-values are adjusted by Holm’s procedure at a nominal 0.05 level; the associated difference CIs are unadjusted 95% intervals. Interpretation is restricted to exploratory within-batch comparisons. Five observations provide limited evidence about distributional assumptions, and a single batch per reinforcement category cannot quantify between-batch variability or distinguish category effects from batch effects.

The ten compression results in each thermal group came from five parent prisms. Because the available specimen identifiers do not retain the pairing of halves to parent prisms, compression means, SDs, CVs, and ranges are reported descriptively, without a confidence interval or hypothesis test that would assume ten independent replicates. The strength plots show means with asymmetric whiskers extending to the observed minimum and maximum. Labels report the mean and the signed deviations from it in MPa; individual data-point markers are omitted. SDs and confidence intervals are reported separately in the accompanying tables.

## 3. Results and Discussion

**Figure 3:**
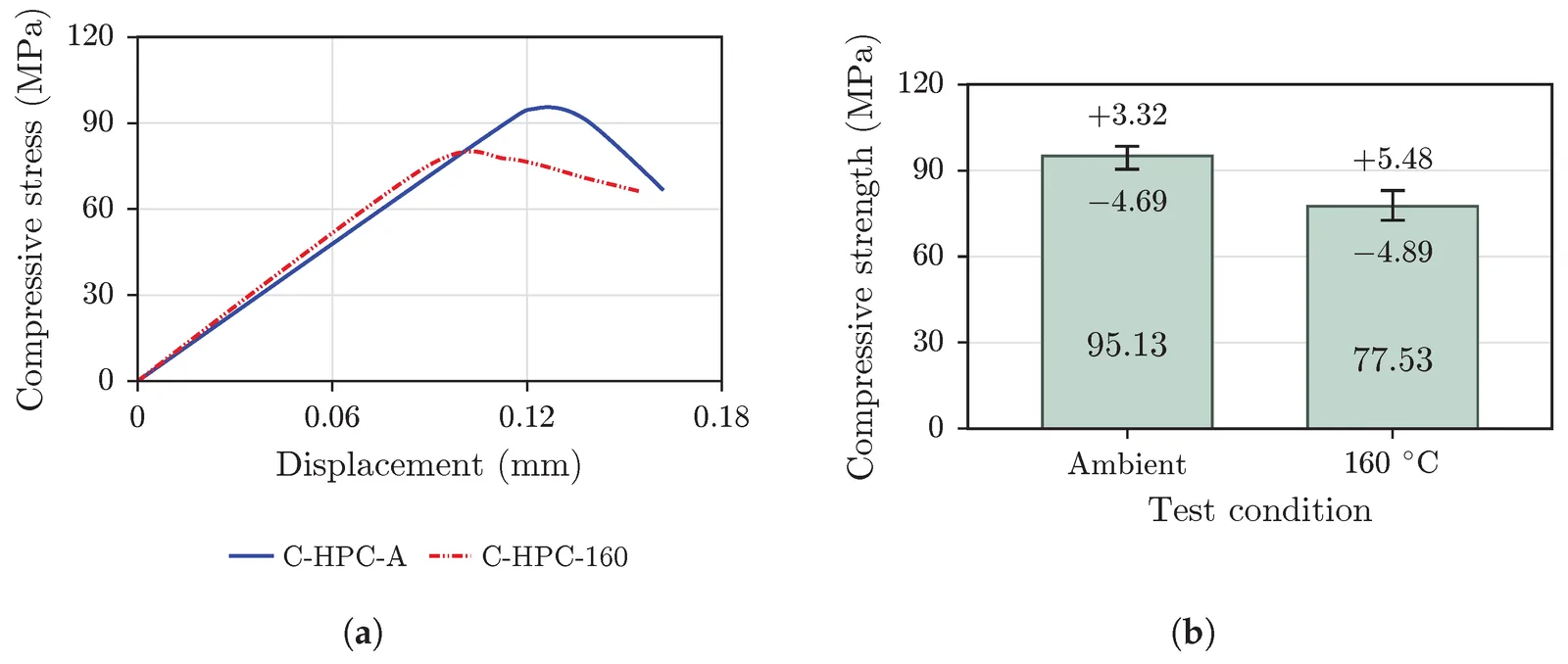
Plain-concrete compression: (**a**) recorded curves; (**b**) mean strengths with minimum–maximum whiskers and signed deviations from the mean (MPa).

**Figure 4:**
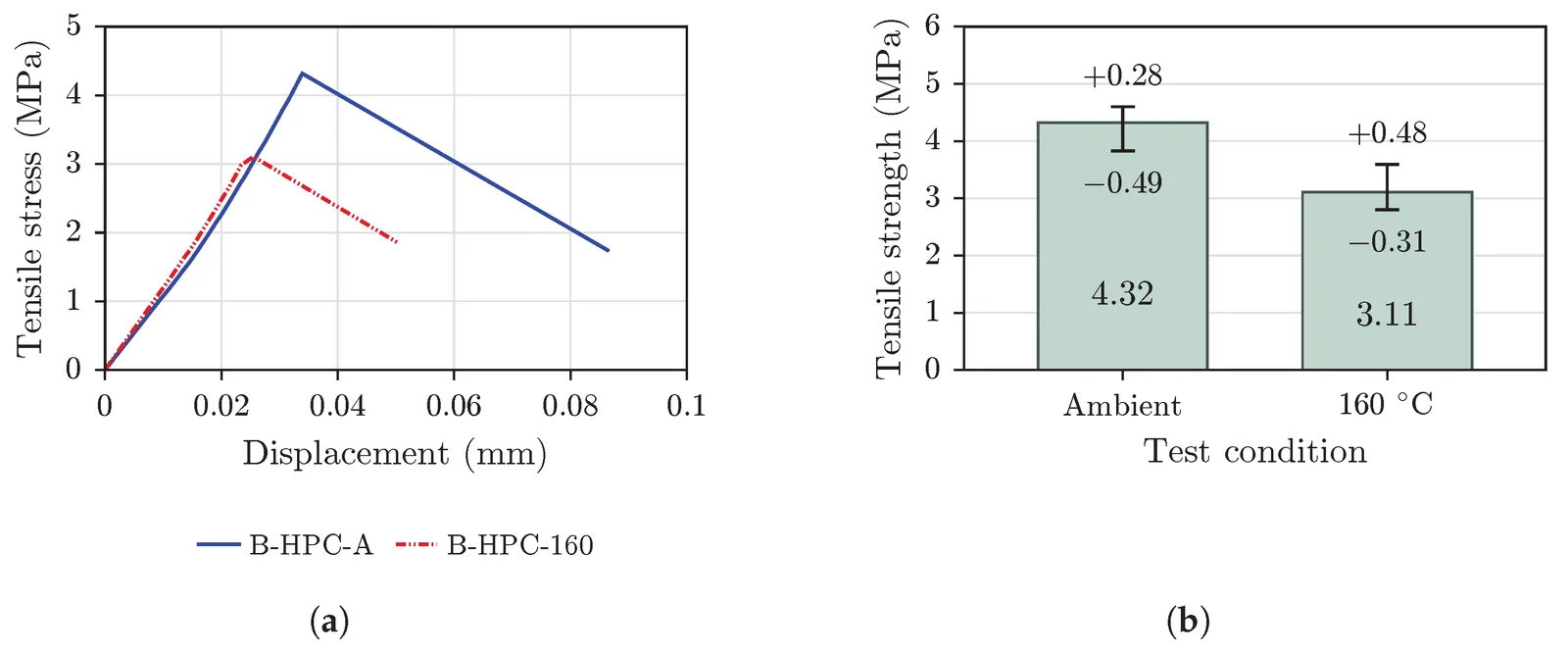
Plain-concrete tension: (**a**) recorded curves; (**b**) mean strengths with minimum–maximum whiskers and signed deviations (MPa).

**Figure 5:**
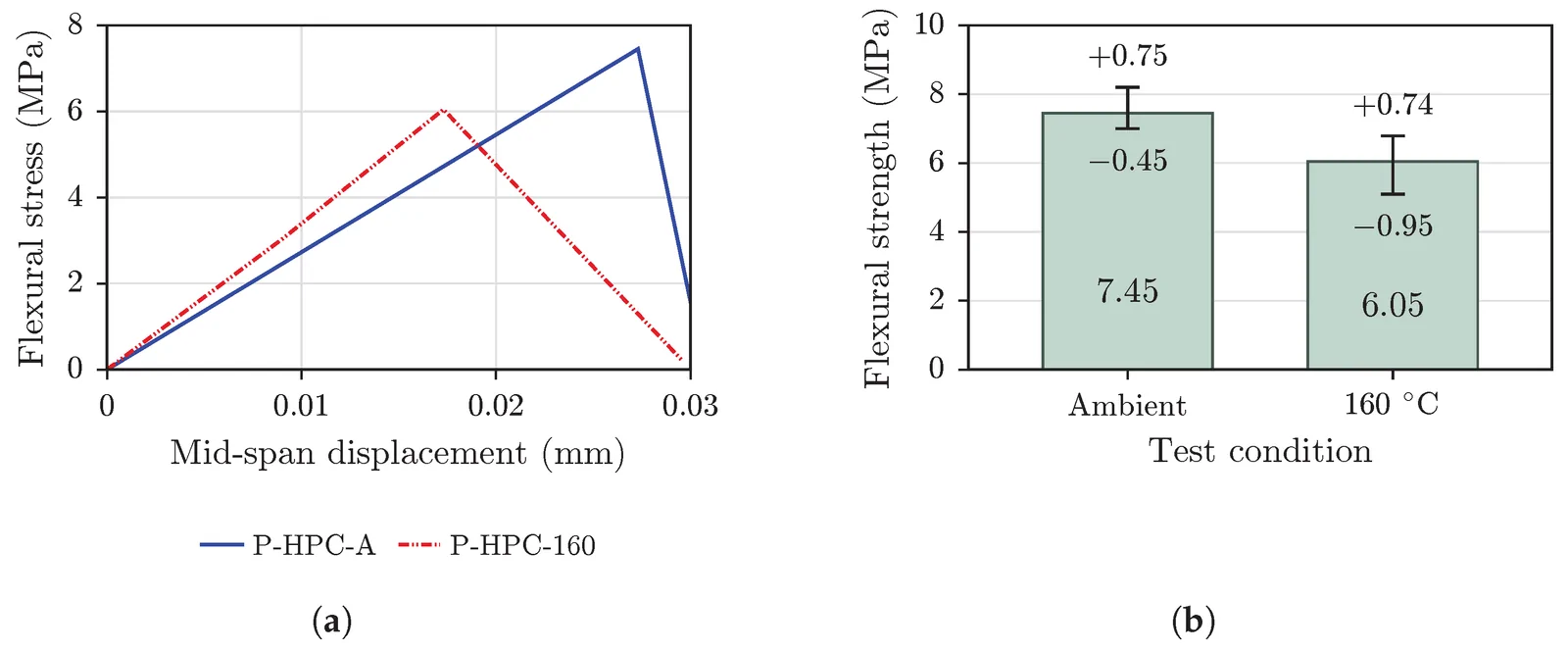
Plain-concrete flexure: (**a**) recorded curves; (**b**) mean strengths with minimum–maximum whiskers and signed deviations (MPa).

**Figure 6:**
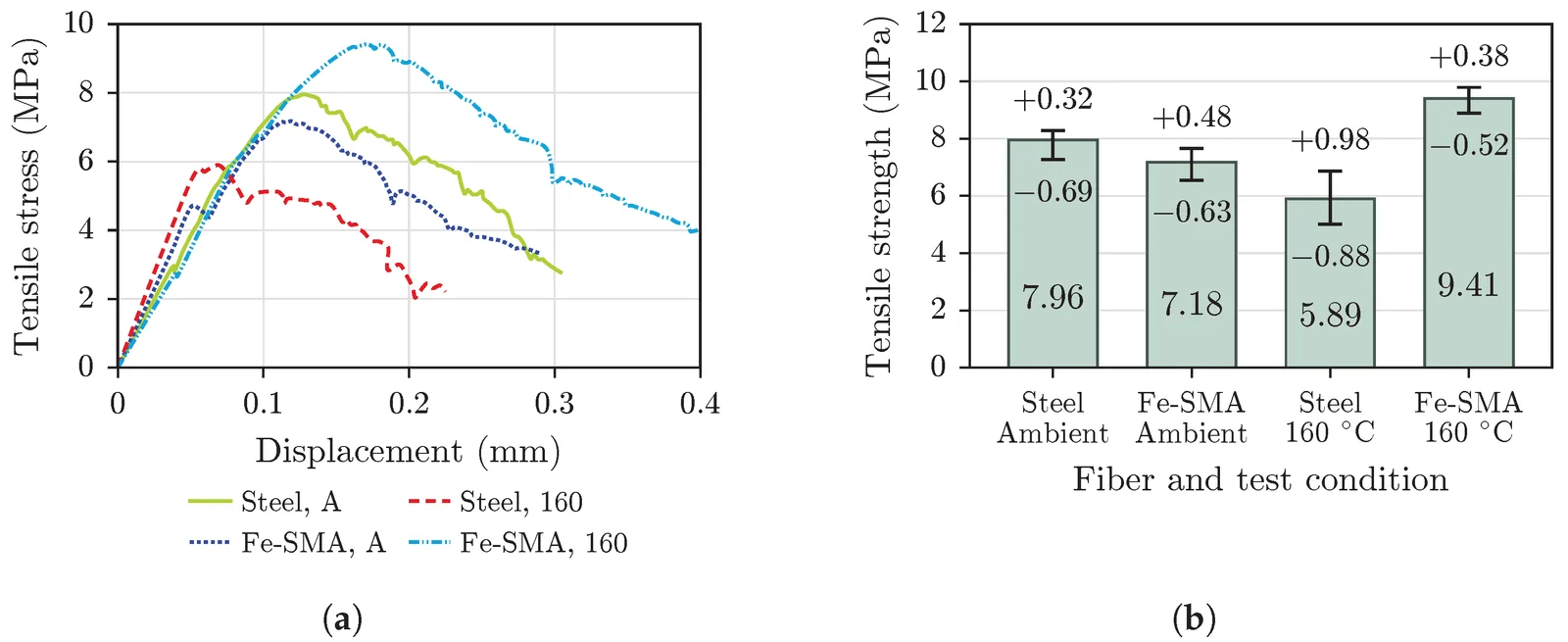
Fiber-reinforced tension: (**a**) recorded curves; (**b**) mean strengths with minimum–maximum whiskers and signed deviations (MPa). A: ambient; 160: heated and cooled.

Figure 3, Figure 4, Figure 5 and Figure 6 compare the recorded response curves with mean specimen peak strengths. Table 4, Table 5, Table 6 and Table 7, placed beneath the corresponding figures, report sample sizes, means, SDs, CVs, observed ranges, and applicable confidence intervals. The bars show the arithmetic mean; their whiskers extend to the observed minimum and maximum. The signed labels give the lower and upper deviations from the mean in MPa. These range bars are not SD bars or confidence intervals. The response-curve panels are retained from the submitted plots and are not the source of the statistical summaries.

Mean compressive strength decreased from 95.13 to 77.53 MPa after heating and cooling, a reduction of 18.5%. The SD increased from 2.95 to 3.33 MPa and the CV from 3.10% to 4.30%, indicating greater relative within-batch scatter in the heated condition. These summaries are descriptive because the compression results are paired halves from five parent prisms. The strength loss is consistent with thermal alteration of the matrix, although moisture loss and microcracking were not measured separately.

Mean plain-concrete tensile strength decreased from 4.32 to 3.11 MPa, a reduction of 28.0%. The CV increased from 7.61% to 12.82%, while the within-batch 95% CI for the heated mean was 2.61–3.61 MPa. The heated-minus-ambient difference was −1.21 MPa (95% CI −1.75 to −0.67 MPa). The larger relative reduction in tension than in compression indicates greater sensitivity of the tensile response under this conditioning regime.

Mean flexural strength decreased from 7.45 to 6.05 MPa, a reduction of 18.8%. The SD increased from 0.49 to 0.73 MPa and the CV from 6.64% to 12.01%. The mean difference was −1.40 MPa (95% CI −2.33 to −0.47 MPa). The recorded peak also occurred at a smaller displacement after heating; without a local strain measurement, this is a displacement observation rather than a quantified ductility index.

Across the plain-concrete tests, mean strength reductions ranged from 18.5% to 28.0%. Thus, the thermal treatment affected the matrix as well as activating the Fe-SMA fibers. The plain and steel-fiber controls help interpret the net composite response, but do not separately measure prestress or changes at the fiber–matrix interface.

Mean steel-fiber tensile strength decreased from 7.96 to 5.89 MPa, while its CV increased from 5.07% to 13.99%. In contrast, the Fe-SMA mean increased from 7.18 to 9.41 MPa (31.1%), and its CV decreased from 5.78% to 3.82%. The heated Fe-SMA group therefore combined higher mean strength with lower relative within-batch scatter in this dataset. Its strength increase was 2.23 MPa (95% CI 1.66–2.80 MPa; Holm-adjusted p=0.000079). The small samples and single batch per material limit generalization of these variability patterns.

The ambient steel-Fe-SMA contrast may partly reflect different elastic moduli, but the interface and the use of separate material batches prevent assigning it to stiffness alone. After heating and cooling, the Fe-SMA group gained strength despite the losses in both control materials. This supports a net activation benefit for the composite without identifying the stress carried by each fiber. The approximately 360 MPa input used in the simulation comes from earlier constrained-fiber tests, as detailed in Section 4.2.2.

Table 8 summarizes the four thermal contrasts. All remained significant at the nominal 0.05 level after Holm adjustment. The difference CIs are unadjusted pointwise intervals; the tests and intervals describe the tested batches and do not establish between-batch reproducibility.

**Figure 7:**
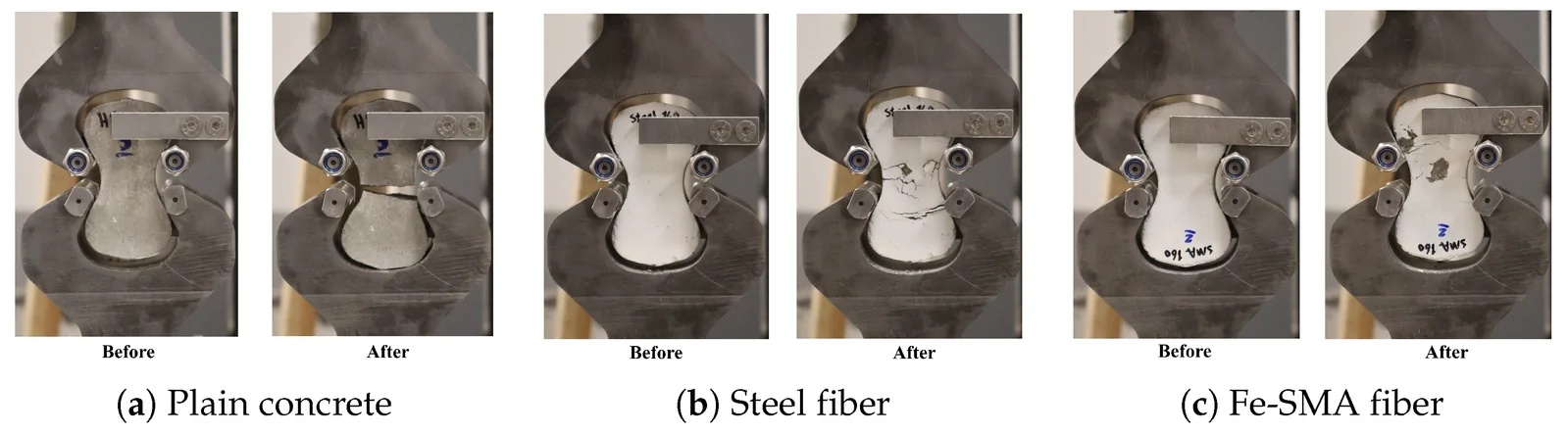
Representative photographs for each specimen type, divided by fiber material, recorded at the start and at the end of the tensile test.

Figure 7 provides macroscopic evidence of the tensile failure patterns. The plain-concrete specimen shows an open, dominant transverse crack and separation of the two parts. In the fiber-reinforced specimens, visible cracking is distributed over a wider region, with branching and local surface detachment, while the specimen retains greater continuity at the end of the recorded test. These features are consistent with the residual load carried after cracking. However, the images do not resolve individual fiber surfaces, interface damage, or the proportions of fibers that pulled out or ruptured. The different termination conditions also prevent direct quantitative comparison of final crack opening. The photographs should therefore be interpreted qualitatively, without treating them as microscopic evidence.

Previous pull-out studies show that geometry and thermal treatment influence Fe-SMA bond response [27,31]. These mechanisms provide context for interpreting crack bridging, but adhesion loss, friction, and matrix damage cannot be separated from the present photographs. Neither SEM nor optical microscopy of raw fibers or fracture surfaces was performed. Microscopic characterization with calibrated scale bars and systematic fracture-surface sampling is consequently identified as a priority for future work.

## 4. Computational Modeling and Parametric Analysis

The ABAQUS/CAE analysis connects the measured briquette response to two numerical parameter sweeps. The geometry and random straight-fiber placement are described in Section 4.1. Section 4.2 separates the matrix calibration against the present tests from the Fe-SMA inputs adopted from earlier characterization [25,26]. Loading restraints and the full-bond idealization are specified in Section 4.3. Mesh refinement is assessed against the measured global response in Section 4.4 before fiber content and diameter are varied separately in Section 4.5. Agreement with these measurements checks the model within the investigated configuration; it does not independently establish the local interface law.

### 4.1. Generation of the Random Distribution of Fibers

**Figure 8:**
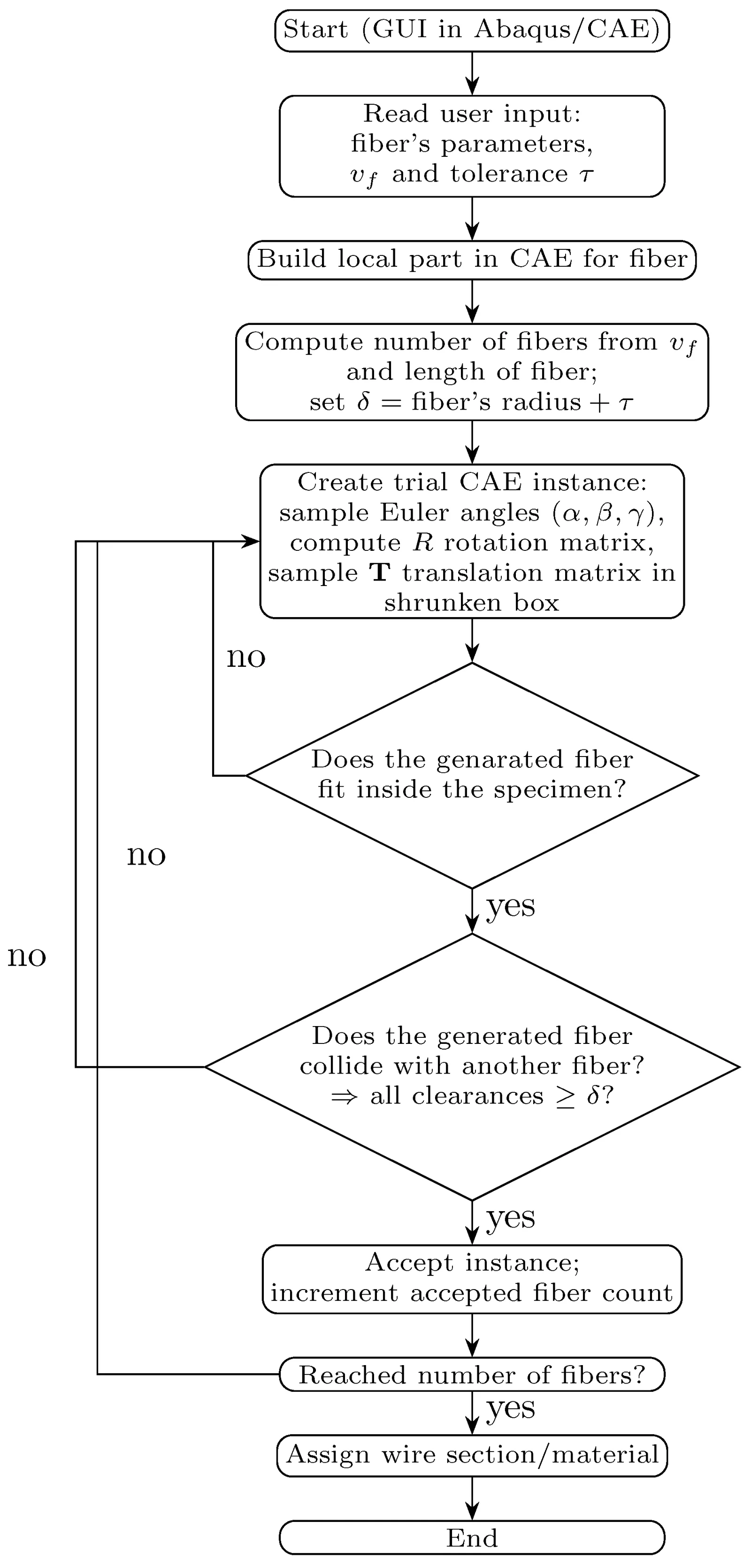
Briquette geometry and checks used to generate the random straight-fiber layout.

**Figure 9:**
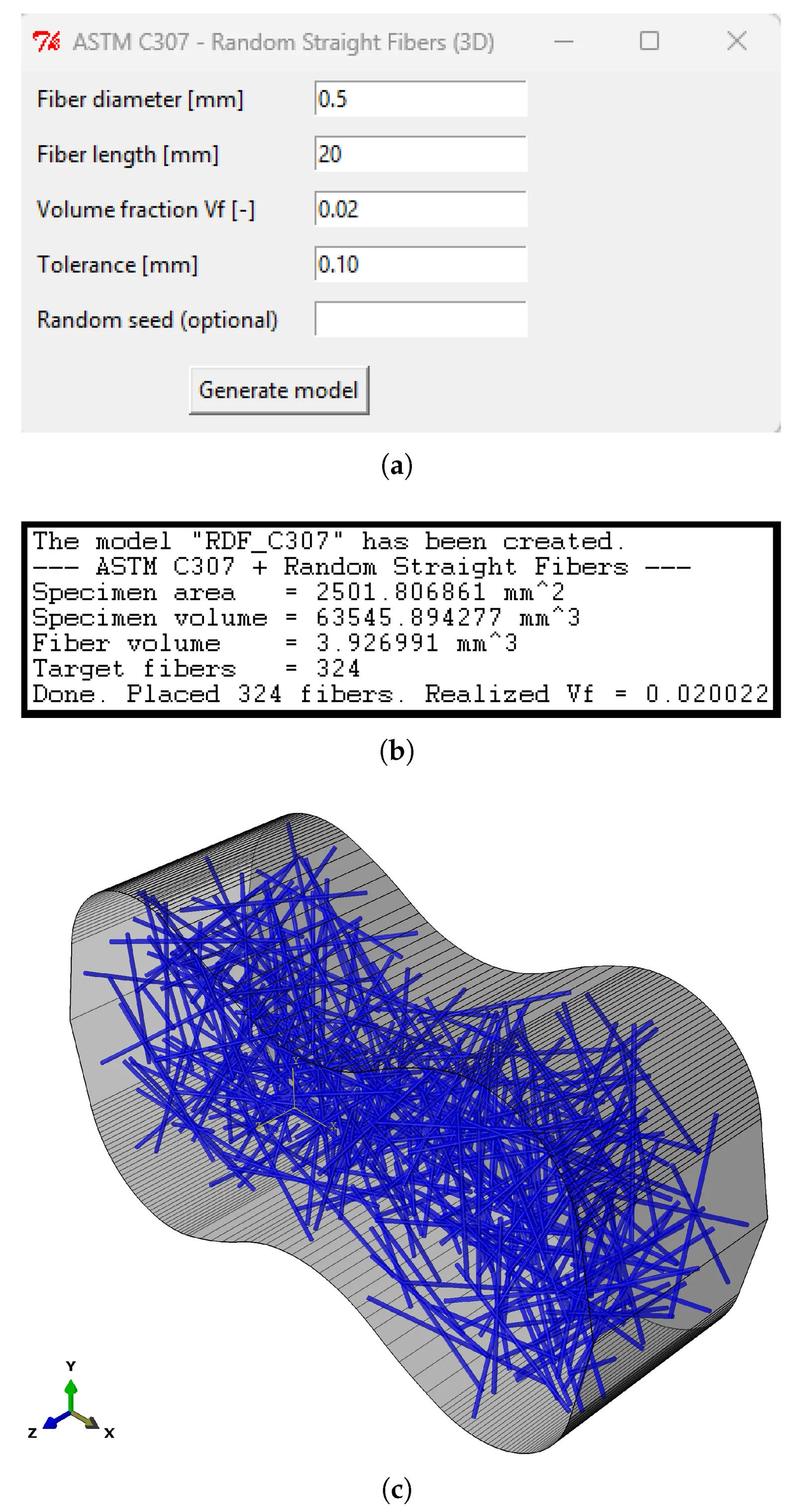
ABAQUS/CAE interface for generating the briquette reinforcement: inputs, completion message, and resulting fiber layout. (**a**) GUI of the developed algorithm in ABAQUS/CAE. (**b**) Communication about the completion of the process. (**c**) Final result in ABAQUS/CAE.

A Python (v. 3.14) script integrated into ABAQUS/CAE generated the random fiber distribution within the three-dimensional briquette. Its workflow and graphical interface are shown in Figure 8 and Figure 9.

The script places trial fibers within the specimen and checks boundary containment and interference with previously accepted fibers. User inputs include fiber diameter, length, target volume fraction, and clearance tolerance. The resulting realization provides the discrete fiber layout for the finite-element model. Figure 9 shows the input interface, completion notification, and generated geometry.

### 4.2. Constitutive Models of Materials

#### 4.2.1. Concrete Damage Plasticity Model

Concrete damage plasticity (CDP) supplies the matrix constitutive law, following Lubliner et al. [32] and Lee and Fenves [33]. In its uniaxial form, tensile and compressive damage scale the elastic stress calculated from the recoverable strain. Equations (1) and (2) state these standard relations and fix the notation used here; they are not new constitutive equations.

(1) $$\sigma_{t}=\left(1-d_{t}\right)E_{0}\;\left(\varepsilon_{t}-{\tilde{\varepsilon }}_{t}^{pl}\right)$$

(2) $$\sigma_{c}=\left(1-d_{c}\right)E_{0}\;\left(\varepsilon_{c}-{\tilde{\varepsilon }}_{c}^{pl}\right)$$

The subscripts *t* and *c* distinguish tension from compression. Total strain is denoted by ε, its equivalent plastic part by ${\tilde{\varepsilon }}^{pl}$, and the undamaged modulus by $E_{0}$. The factors $1-d_{t}$ and $1-d_{c}$ express stiffness retention. The equivalent plastic strains in these equations differ from the cracking and inelastic strains used in the tabulated CDP inputs.

Both thermal states use the same CDP shape and regularization settings: eccentricity ϵ=0.1, biaxial-to-uniaxial strength ratio $\sigma_{b0}/\sigma_{c0}=1.16$, $K_{c}=0.67$, dilation angle 37°, and viscosity $1\times 10^{-10}$. State-specific stiffness and strength represent the measured thermal deterioration. The ambient matrix uses $E_{c}=41.5$ GPa, $f_{c}=95.1$ MPa, and $f_{t}=4.3$ MPa; the cooled matrix after the 160 °C treatment uses 38.5 GPa, 77.5 MPa, and 3.1 MPa, respectively.

Matrix calibration used four plain-concrete models: one briquette and one compression cube for each thermal state. Their imposed loading and restraints reproduced the corresponding tests. The tensile and compressive input curves were adjusted against Figure 3 and Figure 4, respectively. Fibers were absent from these calibration models, so the fitted matrix response was established before incorporating reinforcement.

**Figure 10:**
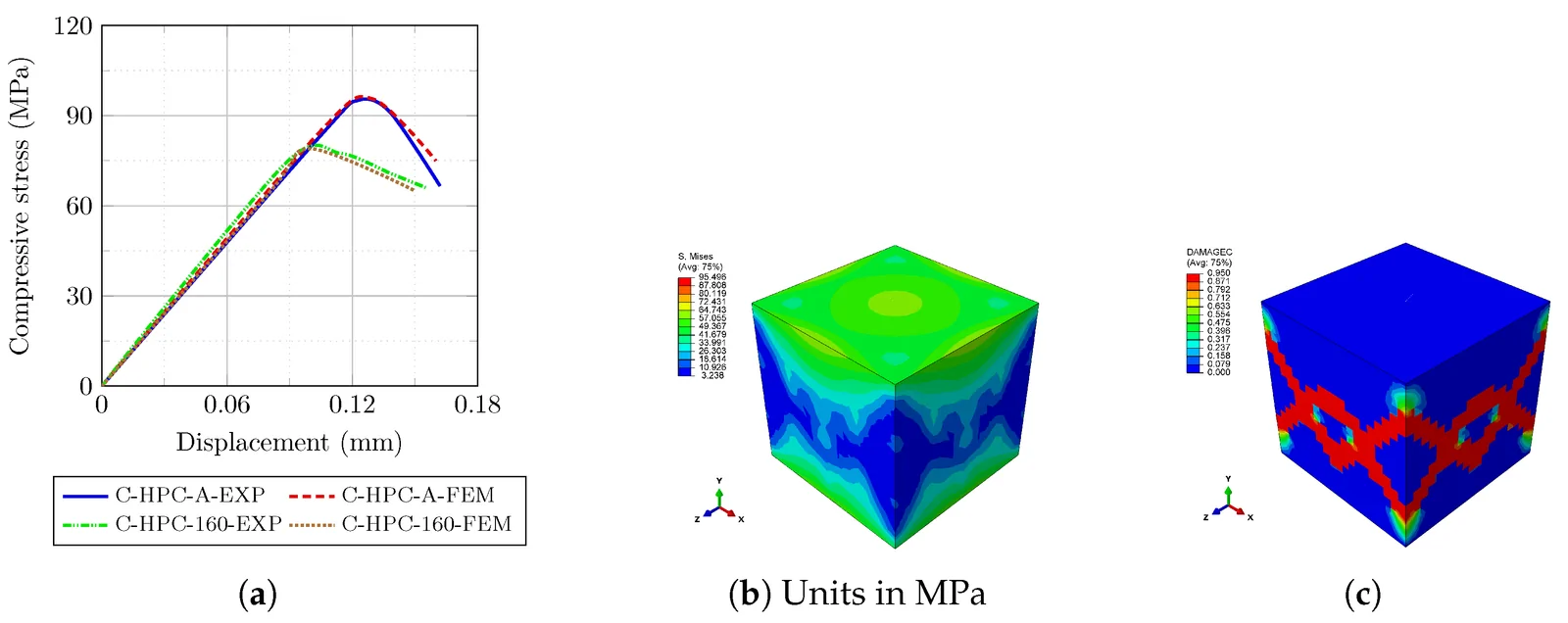
Compression calibration for the two matrix states: (**a**) calculated and measured curves; (**b**) computed stress field; (**c**) compressive damage field.

**Figure 11:**
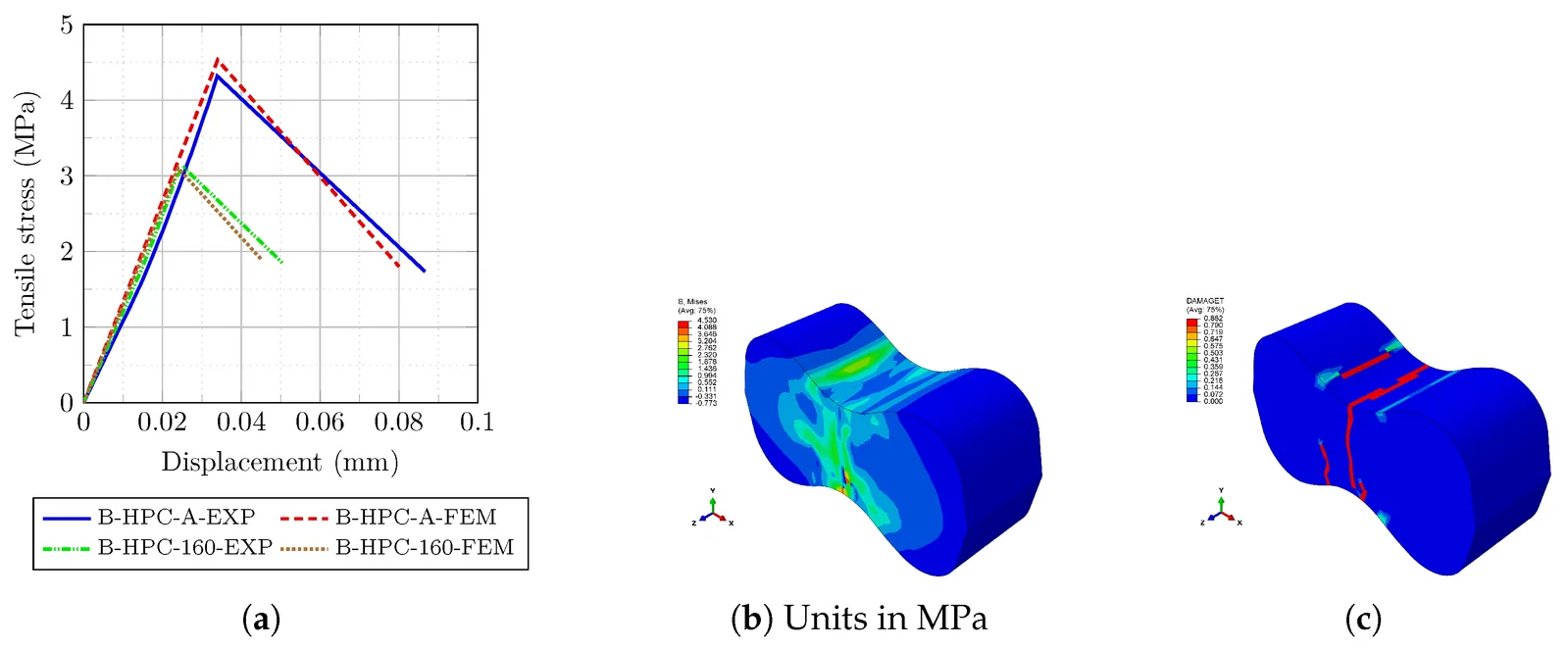
Tension calibration for the two matrix states: (**a**) calculated and measured briquette curves; (**b**) computed stress field; (**c**) tensile-damage field.

Figure 10 and Figure 11 compare the calculated compression and tension responses with the curves used to calibrate the matrix model. The agreement demonstrates calibration consistency; it is not an independent validation against a separate experimental dataset.

**Figure 12:**
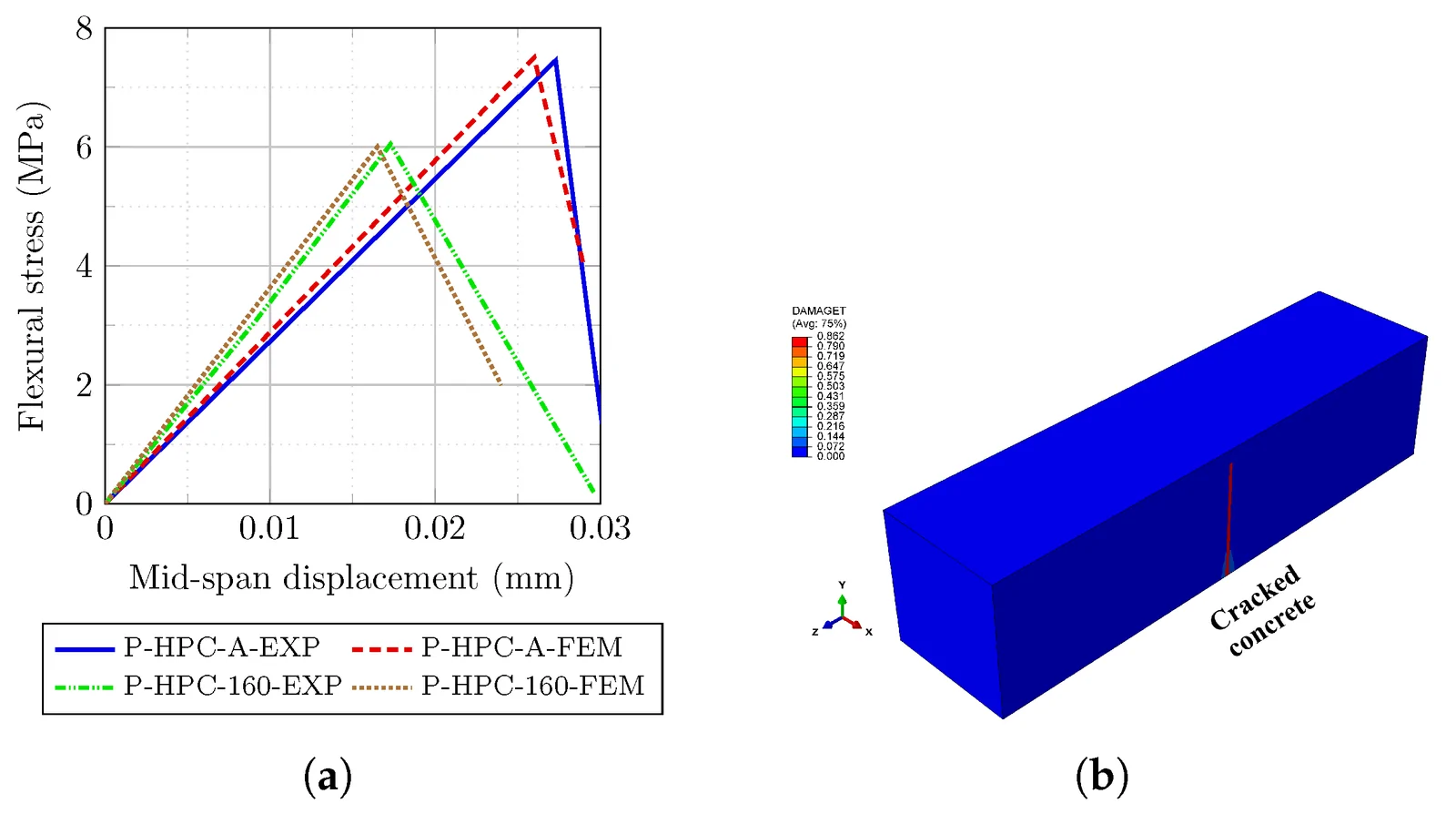
Flexural check of the calibrated matrix inputs: (**a**) comparison with the measured prism response; (**b**) computed tensile damage.

Two further simulations applied the calibrated matrix definitions to plain-concrete prisms in bending, one for each thermal state. Figure 12 compares the resulting flexural curves with the experiments. This provides a check under a different stress distribution within the same test program and supports using these matrix inputs in the reinforced-briquette analysis.

#### 4.2.2. Fe-SMA Material Model

The recovery-stress input is taken from the previously published same-batch experiments [25], rather than from an additional test in this campaign. In that characterization, prestrained 0.5 mm fibers were held at both ends and initially loaded to approximately 100 MPa, limiting compression during heating. A cycle to 160 °C and back to room temperature left a constrained tensile stress of about 360 MPa. This value incorporates the preload and restraint history and describes the cooled fiber. It does not measure the present embedded-fiber stress or the stress during the 150 min chamber hold. The approximately 350 MPa reported in [27] provides related context, but its different material history prevents treating the two tests as equivalent.

**Figure 13:**
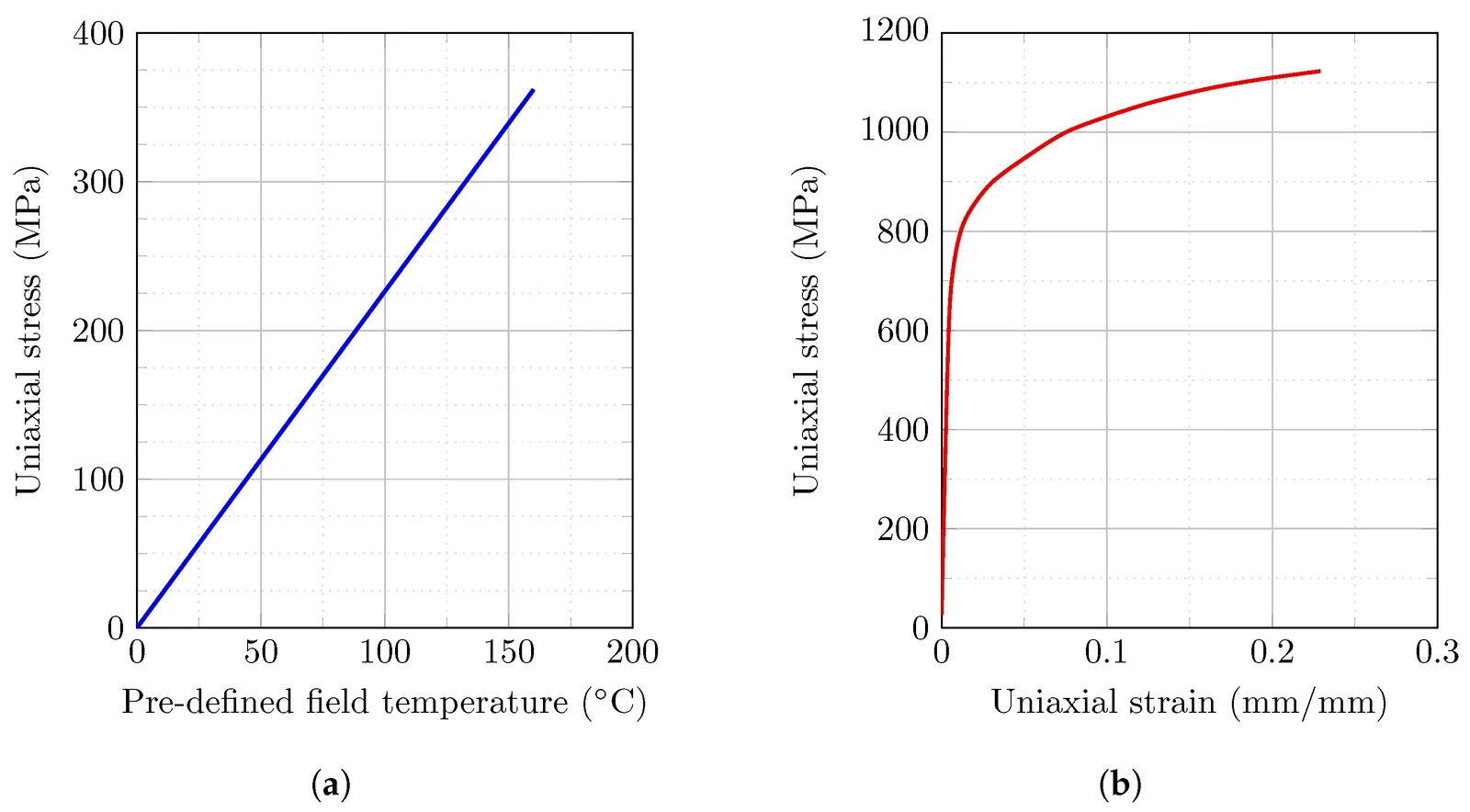
Fe-SMA constitutive approximation: (**a**) effective numerical activation path targeting the residual constrained stress after a 160 °C heating–cooling cycle; (**b**) adopted post-activation mechanical law. These panels show model definitions, not new experimental fiber measurements.

Activation is represented by prescribing fiber contraction through an effective negative thermal-expansion coefficient, an equivalent approach used in earlier numerical studies [34,35]. Figure 13a checks that this imposed loading reaches the approximately 360 MPa constrained target. Its linear path is a numerical activation coordinate, not a measured thermal trajectory: expansion, phase transformation, and cooling are not resolved individually. The target approximates the residual constrained stress from the earlier full cycle. Actual transfer from a short embedded fiber depends on its anchorage and restraint, so local stresses may be lower and nonuniform. The input is therefore a supported constitutive assumption, not a measurement of prestressing inside these briquettes.

Figure 13b defines the adopted isotropic-hardening approximation. Its elastic moduli are 160 GPa before activation and 85 GPa afterward; the remaining inputs are Poisson’s ratio 0.275, a 500 MPa yield stress, and a final tabulated point of 1125 MPa at strain 0.225. These modeling choices are distinguished from the earlier measured ultimate stress of approximately 1150 MPa and failure strain of about 30% [25]. No standalone tensile or recovery-stress test of the fibers was added in the present campaign.

### 4.3. Boundary Conditions and Interactions

**Figure 14:**
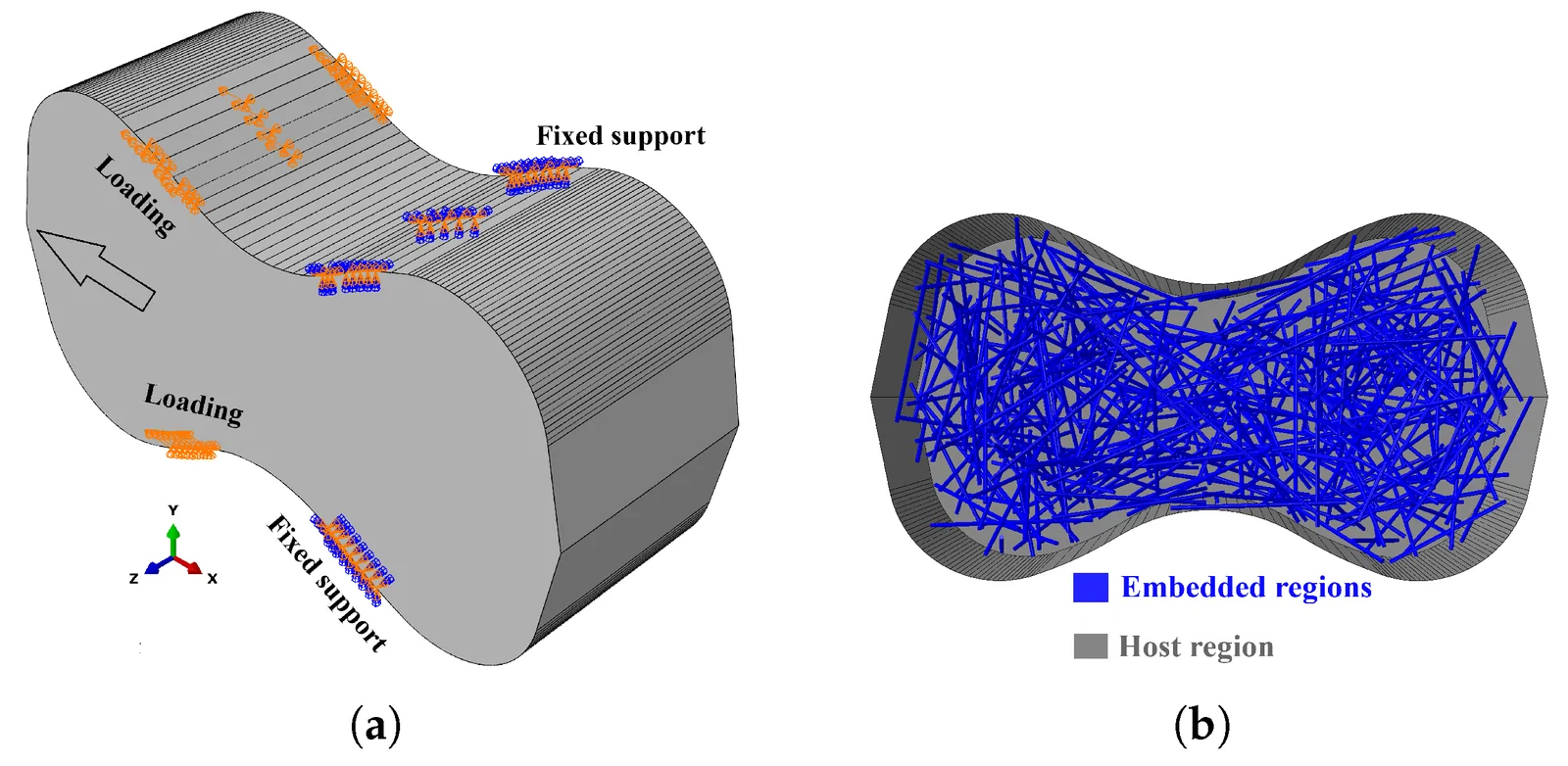
(**a**) Applied boundary conditions, and (**b**) interaction between fibers and specimen.

The mesh combines a deformable solid for the concrete with truss elements for the straight fibers. Restraints on the surfaces contacting the two supports reproduce the test arrangement in Figure 2. At the opposite end, two asymmetrically arranged loading surfaces receive the prescribed global *x* displacement (U1); their other translational degrees of freedom are fixed. Figure 14a identifies the locations of these constraints.

The model used a predefined field to impose effective fiber contraction and select material properties for the thermally conditioned state. This procedure represents the residual mechanical effect of activation and matrix conditioning, rather than a heat-transfer simulation of the measured chamber cycle. As shown in Figure 14b, the embedded-region constraint ties the fiber translational degrees of freedom to the surrounding concrete, enforcing full displacement compatibility and suppressing relative slip.

Perfect bond was adopted as an idealization for comparison of the global briquette response and for the exploratory parameter sweeps. Agreement with the observed response supports its use within the calibrated configuration, but does not establish that the physical interface remains perfectly bonded. The assumption is most defensible before substantial debonding and becomes restrictive after cracking, particularly for straight short fibers. It idealizes efficient prestress and load transfer; it is not a rigorous upper bound on every response quantity. An explicit bond-slip law would permit anchorage-dependent recovery-stress transfer, progressive debonding, and frictional pull-out. Relative to the present model, these processes could reduce effective reinforcement stiffness, alter the peak load, and change post-peak load and energy dissipation. Their magnitude and even the detailed post-peak curve shape require interface calibration. Fiber-diameter trends may also change because anchorage efficiency is not constant across diameters. A coupled activation and bond-slip model, calibrated using pull-out data, is needed to assess these effects quantitatively.

### 4.4. Mesh Sensitivity Analysis and Model Validation

**Figure 15:**
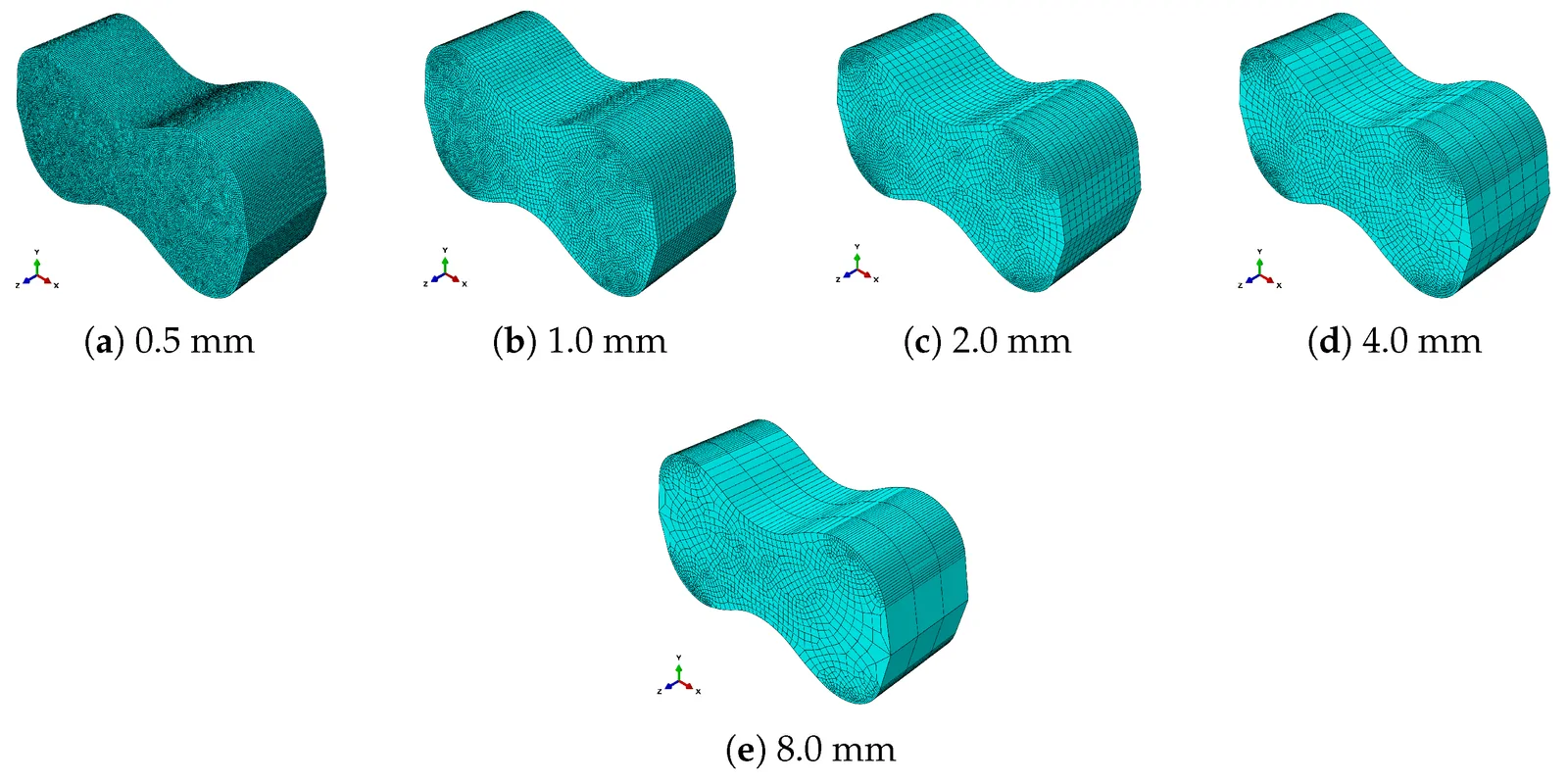
Briquette discretizations compared in the mesh-refinement study.

**Figure 16:**
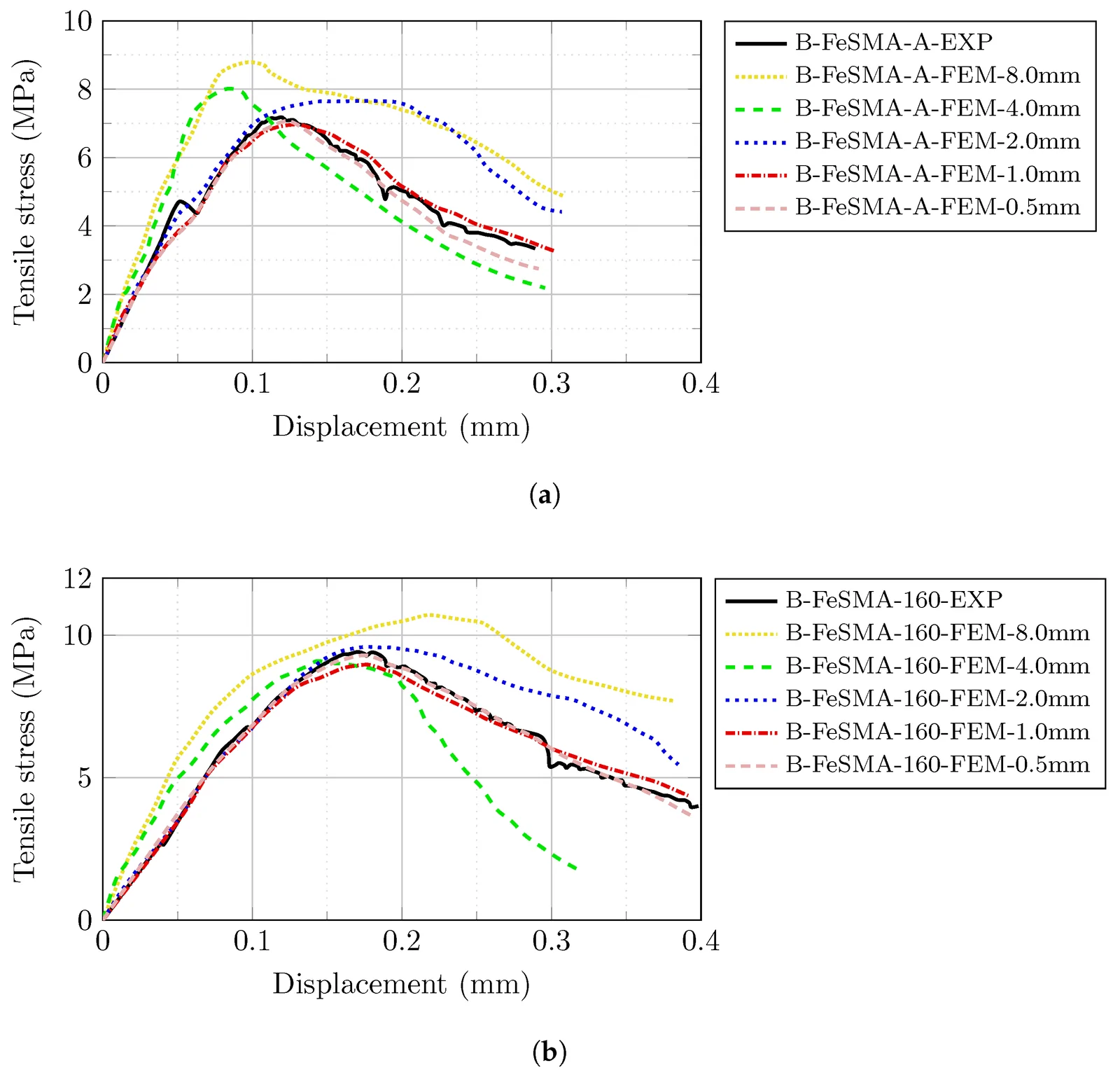
Mesh-refinement curves compared with the briquette experiments: (**a**) ambient condition; (**b**) after heating and cooling.

**Figure 17:**
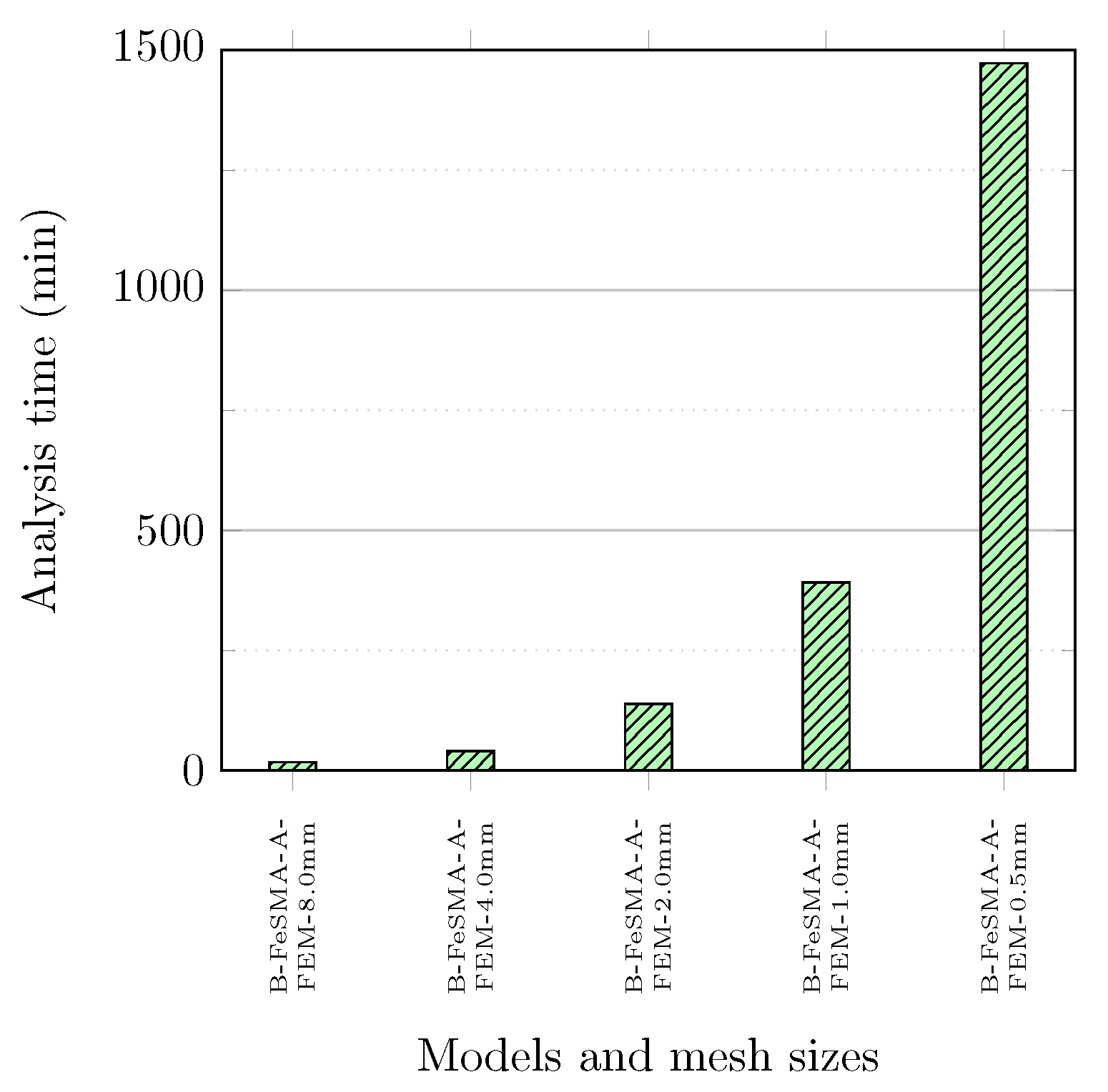
Comparison between models’ analysis time.

Five nominal element sizes were compared: 8.0, 4.0, 2.0, 1.0, and 0.5 mm. Their discretizations appear in Figure 15, and the resulting tensile curves and average run times in Figure 16 and Figure 17. The analyses used a Ryzen 7-4800 processor (by AMD, Santa Clara, CA, USA) running at 2.90 GHz with 64 GB RAM. Accuracy and computational demand were considered together when selecting the working mesh.

Neither the 8 mm nor the 4 mm mesh reproduced the observed stiffness and subsequent nonlinear response satisfactorily. At 2 mm, the elastic branch improved, but substantial nonlinear differences remained. The 1.0 mm and 0.5 mm solutions captured the principal curve features. Refinement from 1.0 to 0.5 mm produced only a small further change while increasing the run time by approximately a factor of four, so the parameter study used 1.0 mm. This is a practical discretization choice for the present model, rather than evidence of a mesh-independent constitutive response.

**Figure 18:**
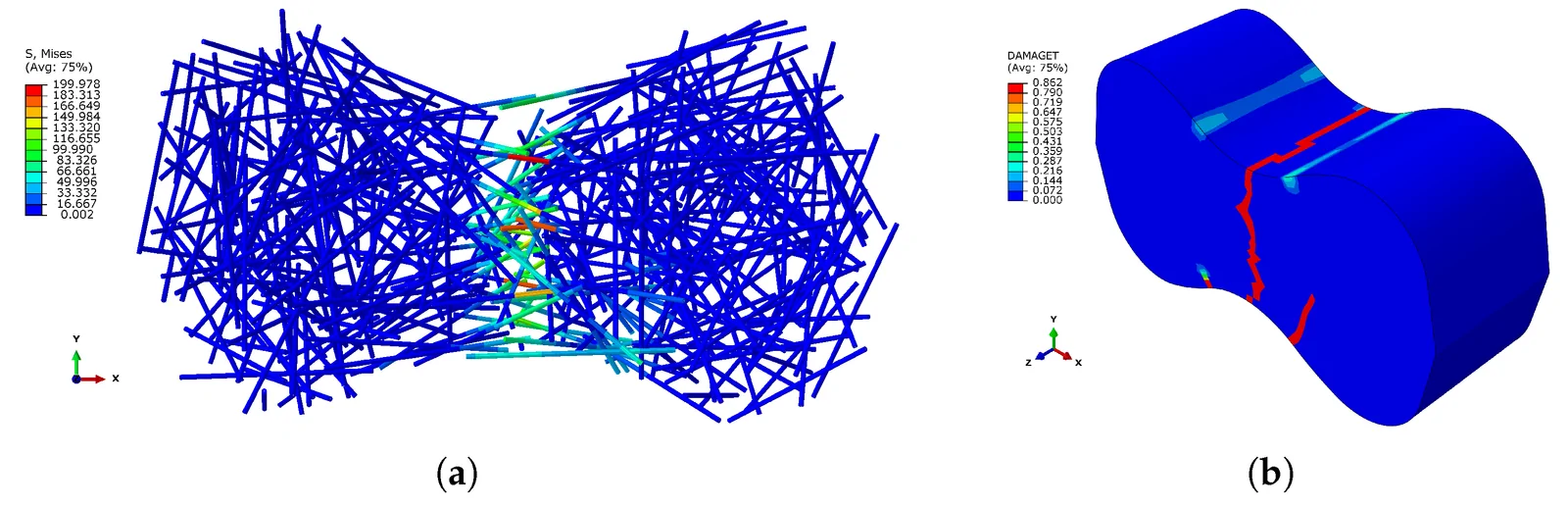
Predicted internal response: (**a**) fiber stress field; (**b**) matrix tensile damage.

Figure 16 indicates that the refined models reproduce the principal features of the measured global response. The baseline heated model reaches approximately 9.6 MPa, close to the revised specimen-mean strength of 9.41 MPa and within its within-batch 95% CI of 8.96–9.86 MPa. This comparison concerns global response, not independent identification of the interface law. Figure 18 shows the computed fiber stresses and concrete tensile-damage distribution.

### 4.5. Parametric Analysis

**Figure 19:**
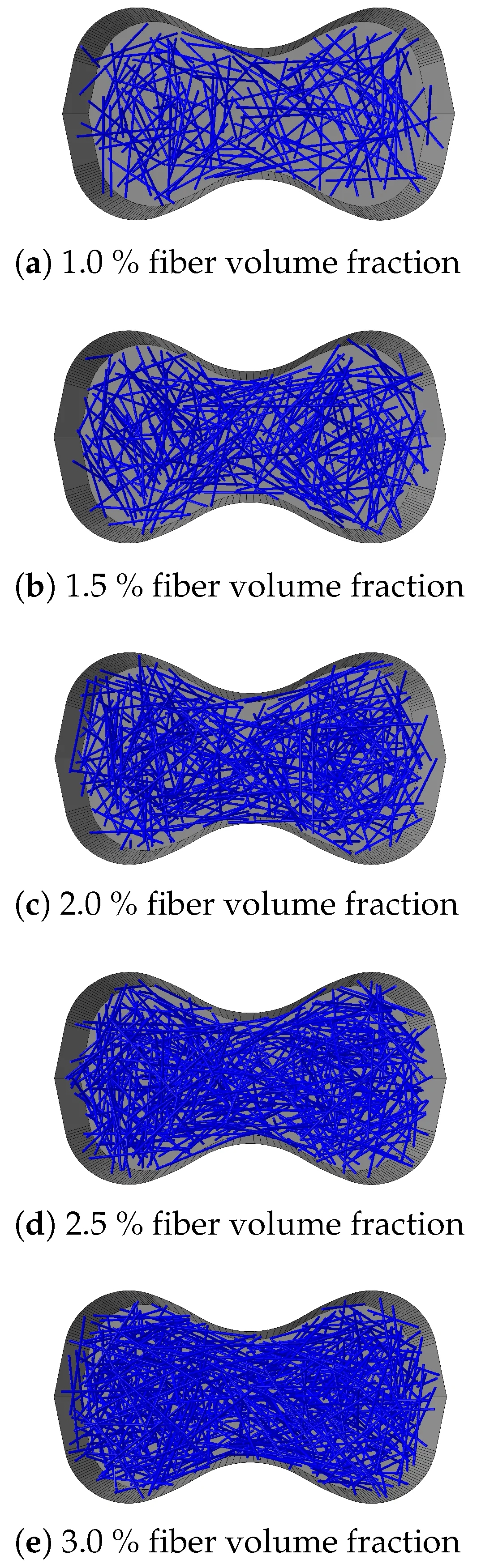
Random fiber configurations for the content sweep; diameter remains 0.5 mm.

**Figure 20:**
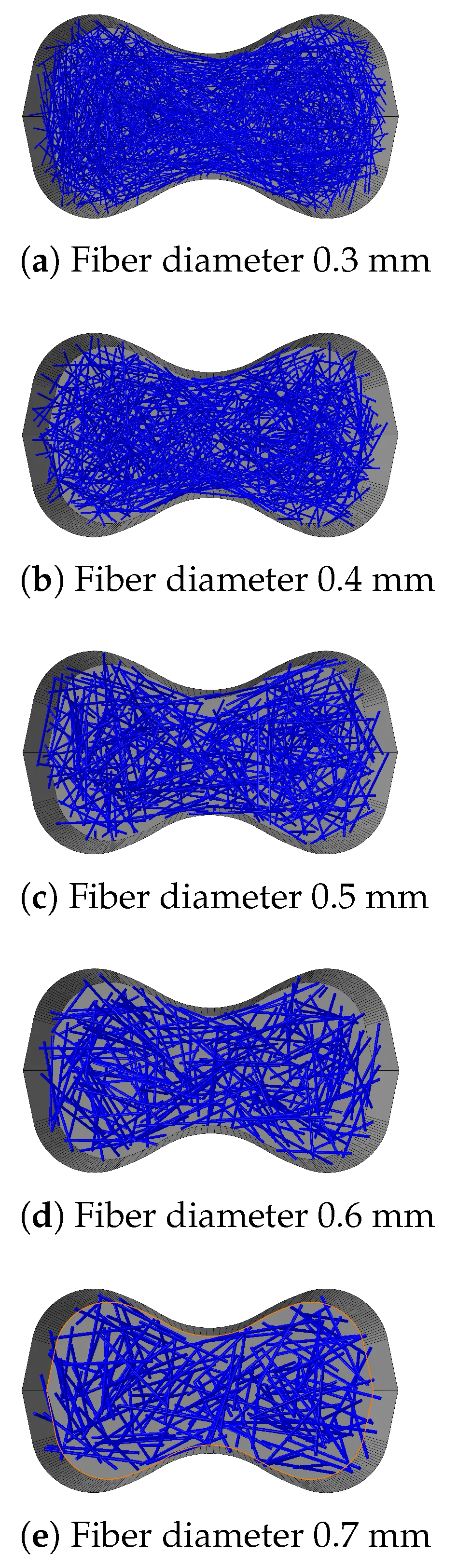
Random fiber configurations for the diameter sweep; content remains 2.0%.

The numerical baseline contains 2.0% fibers of 0.5 mm diameter. Starting from this configuration, one sweep changes content from 1.0% to 3.0% at unchanged diameter (Figure 19); the other changes diameter from 0.3 to 0.7 mm while retaining 2.0% content (Figure 20). Each case represents the mechanically conditioned state associated with 160 °C activation. These are separate sweeps, not a joint search over all diameter–content combinations. Random fiber placement introduces realization dependence, so the curves indicate trends for the generated layouts rather than unique strengths for each nominal configuration.

**Figure 21:**
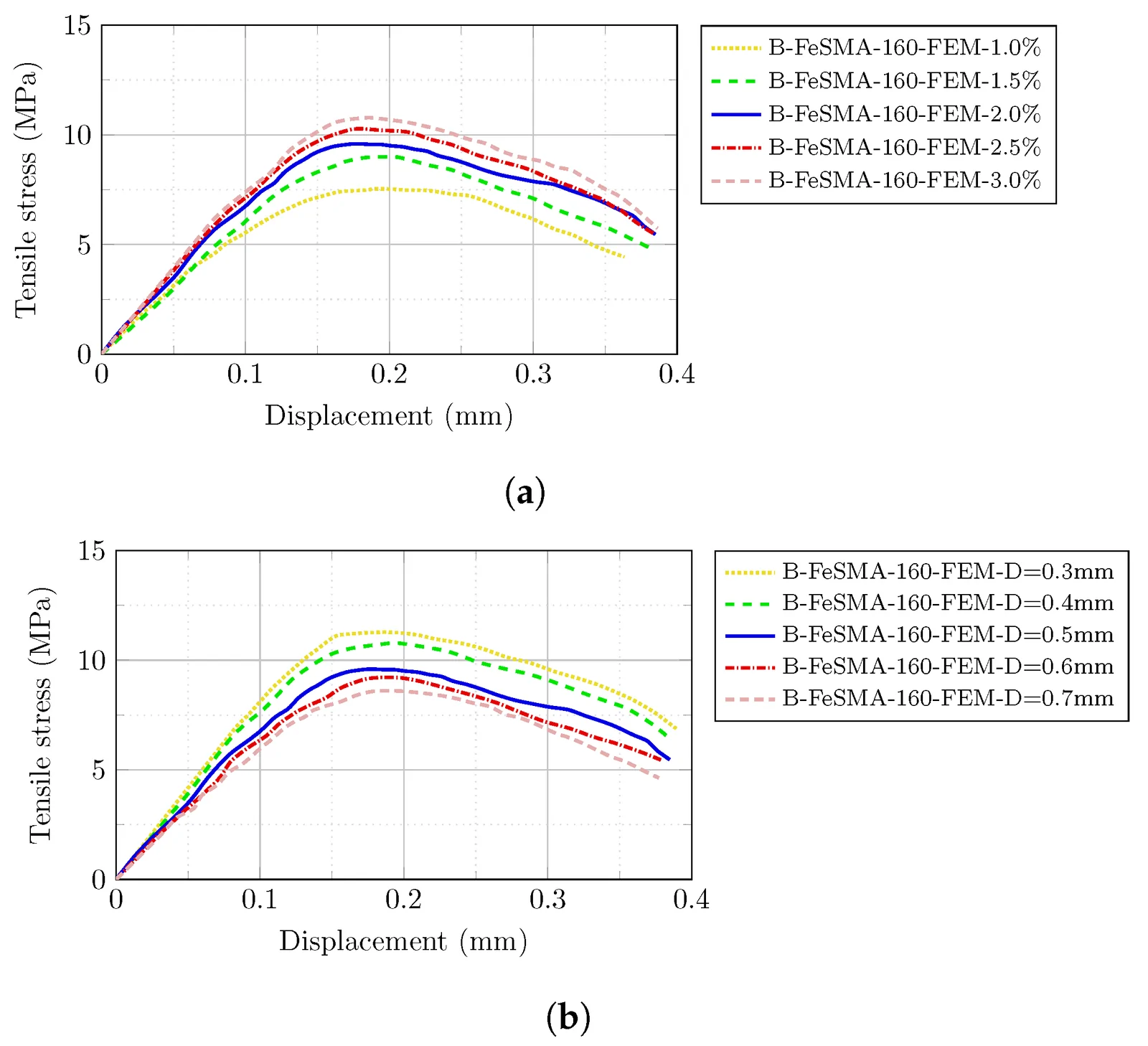
Predicted tensile curves for the separate sweeps: (**a**) 1.0–3.0% content at 0.5 mm diameter; (**b**) 0.3–0.7 mm diameter at 2.0% content.

The volume-fraction sweep in Figure 21a predicts an increase in peak strength from approximately 7.5 MPa at 1.0% to 10.8 MPa at 3.0%, with diameter fixed at 0.5 mm. The 2.0% baseline reaches approximately 9.6 MPa. Thus, 3.0% gives the highest strength within this sweep. The results do not establish a universal minimum content of 2.0%, nor a cost optimum: cost and fresh-state performance were not quantified.

At a fixed volume fraction of 2.0%, the diameter sweep in Figure 21b predicts peak strengths of approximately 11.3 MPa at 0.3 mm and 8.6 MPa at 0.7 mm. For equal fiber length and specimen volume, fiber number varies inversely with the square of diameter; therefore, decreasing diameter distributes the same reinforcement volume over more fibers. The numerical trend is consistent with more distributed reinforcement, but the embedded constraint does not explicitly model the accompanying changes in bond-slip or anchorage. The predictions should therefore be tested experimentally before being used as design recommendations.

Table 9 summarizes approximate peaks read from the plotted numerical curves. A length of 20 mm, diameter of 0.3 mm, and content of 2.0% gave the highest predicted strength among the configurations actually analyzed. In the separate content sweep at 0.5 mm diameter, 3.0% performed best. No combined diameter–content study was conducted, and fiber length was not varied. Consequently, a global optimum in size and content has not been established. The tested 20 mm by 0.5 mm fibers at 2.0% remain the experimental reference; the smaller diameter is a candidate for subsequent validation. The recommendation is conditional on the assumed recovery stress, perfect bond, and individual random realizations.

## 5. Conclusions

For the investigated mixture, straight 20 mm by 0.5 mm fibers, 2.0% fiber content, and 150 min hold at a chamber setpoint of 160 °C followed by cooling, the following conclusions are supported.

- Mean plain-concrete compressive, tensile, and flexural strengths decreased from 95.13 to 77.53 MPa, 4.32 to 3.11 MPa, and 7.45 to 6.05 MPa, respectively. The corresponding reductions were 18.5%, 28.0%, and 18.8%.
- Mean steel-fiber tensile strength decreased from 7.96 to 5.89 MPa, whereas Fe-SMA tensile strength increased from 7.18 to 9.41 MPa. The Fe-SMA increase was 31.1%, or 2.23 MPa (95% CI 1.66–2.80 MPa), and remained significant after Holm adjustment across the four thermal comparisons. This supports a net beneficial activation effect within the tested batch despite matrix degradation.
- The fully bonded three-dimensional model reproduced the main global response features using an effective activation law with a constrained residual-stress target of approximately 360 MPa adopted from prior same-batch fiber characterization. A 1.0 mm mesh provided a practical balance between accuracy and computation time. The agreement does not independently validate local prestress transfer or pull-out behavior.
- Among the analyzed cases, the largest predicted peak was approximately 11.3 MPa for 20 mm long, 0.3 mm diameter fibers at 2.0% content. In the separate content sweep with 0.5 mm diameter, the highest value was approximately 10.8 MPa at 3.0%. These are the best-performing investigated cases, not a joint or universal optimum.

These findings extend the earlier flexural studies to a matched-geometry, straight-fiber direct-tension comparison. The experimental tensile dataset and the briquette parameter trends are the contribution of this work; the Fe-SMA processing route and constrained-fiber characterization remain attributed to the earlier publications.

### Limitations and Future Work

The experiments cover one matrix composition, one laboratory fiber geometry and content, and one activation setpoint. One batch per reinforcement category permits assessment of within-batch scatter but not between-batch reproducibility. Compression measurements from the two halves of a prism are dependent subsamples. Independent batch replication, larger specimen groups, and preservation of parent-prism identifiers should be included in subsequent campaigns.

Core temperature and embedded-fiber recovery stress were not measured in the present briquettes. Earlier thermal measurements and same-batch fiber tests support the protocol and adopted stress scale, but do not directly verify local activation under the present restraint and moisture conditions. Future experiments should instrument sacrificial briquettes during heating and cooling and measure recovery-stress transfer under representative embedment conditions. This is also necessary when extending whole-specimen chamber activation to structural dimensions, where heat loss, gradients, and energy demand become more significant.

No quantitative fresh-state flow or fiber-dispersion measurements were made. Neither raw-fiber microscopy nor fracture-surface microscopy was performed, so interpretation is restricted to macroscopic cracking and global mechanical response. Future work should combine flow and dispersion measurements with optical microscopy or SEM of the fibers and fractured interfaces to distinguish debonding, pull-out, fiber rupture, and matrix damage.

The embedded constraint suppresses slip and the effective activation law does not resolve the physical heating–cooling transformation path. Bond-slip sensitivity, calibrated interface laws, multiple random realizations, and a joint fiber-length–diameter–content study are needed before robust optimization. Experimental testing of smaller diameters must also verify attainable recovery stress, anchorage, and castability; economic optimization requires explicit material and processing costs.

## Figures and Tables

**Table 1:** Comparison of the fiber materials and adopted numerical inputs.

Property	Fe-SMA	Steel Reference
Geometry	Straight, 20 mm long, 0.5 mm diameter	Straight, 20 mm long, 0.5 mm diameter
Aspect ratio; volume fraction	40; 2.0%	40; 2.0%
Condition before mixing	Annealed at 850 °C for 2 h; prestrained 5.0%±1.0% before cutting	As supplied; passive reinforcement
Tensile strength	Approximately 1150 MPa in prior same-batch tests	Approximately 1345 MPa, supplier information
Young’s modulus	160 GPa before activation; 85 GPa after activation, adopted model values	210 GPa, supplier information
Recovery stress	Approximately 360 MPa after a constrained 160 °C heating–cooling cycle in prior tests	No shape memory recovery stress assigned
Additional Fe-SMA model inputs	Yield stress 500 MPa; ultimate stress 1125 MPa at strain 0.225; Poisson ratio 0.275	Not used in the Fe-SMA parametric model

Fe-SMA characterization and processing are reported in [24,25]; the tabulated model inputs describe the constitutive approximation, not new fiber tests. The 360 MPa value is a constrained-fiber reference and is not a measured uniform stress in the embedded short fibers.

**Table 2:** Concrete mix design.

Component	Amount in kg/m^3^
Cement II 42.5R	700.0
Limestone powder	300.0
Silica Fume	100.0
Fine sand (0–0.5 mm)	300.0
Coarse sand (0.5–1.0 mm)	710.0
Superplasticizer	16.5
Shrinkage reducing agent	16.5
Water	190.0

**Table 3:** Classification of tested specimens based on the defined parameters.

Geometry	Fiber	Temperature	Label	No. of Specimens
	No fiber		B-HPC-A	5
Briquette	Steel	Ambient	B-Steel-A	5
	Fe-SMA		B-FeSMA-A	5
	No fiber		B-HPC-160	5
Briquette	Steel	160 °C	B-Steel-160	5
	Fe-SMA		B-FeSMA-160	5
Prismatic	No fiber	Ambient	P-HPC-A	5
		160 °C	P-HPC-160	5
Prism halves	No fiber	Ambient	C-HPC-A	10
		160 °C	C-HPC-160	10

**Table 4:** Descriptive statistics for the compressive strengths in Figure 3.

Group	*n*	Mean	SD	CV (%)	Range	95% CI
C-HPC-A	10	95.13	2.95	3.10	90.44–98.45	-
C-HPC-160	10	77.53	3.33	4.30	72.64–83.01	-

All strengths are in MPa. Ten halves came from five prisms; no CI assuming independent halves is reported.

**Table 5:** Descriptive statistics for the plain-concrete tensile strengths in Figure 4.

Group	*n*	Mean	SD	CV (%)	Range	95% CI
B-HPC-A	5	4.32	0.33	7.61	3.83–4.60	3.91–4.73
B-HPC-160	5	3.11	0.40	12.82	2.80–3.59	2.61–3.61

All strength quantities are in MPa. CIs are pointwise intervals for within-batch means.

**Table 6:** Descriptive statistics for the flexural strengths in Figure 5.

Group	*n*	Mean	SD	CV (%)	Range	95% CI
P-HPC-A	5	7.45	0.49	6.64	7.00–8.20	6.84–8.06
P-HPC-160	5	6.05	0.73	12.01	5.10–6.79	5.15–6.95

All strength quantities are in MPa. CIs are pointwise intervals for within-batch means.

**Table 7:** Descriptive statistics for the fiber-reinforced tensile strengths in Figure 6.

Group	*n*	Mean	SD	CV (%)	Range	95% CI
B-Steel-A	5	7.96	0.40	5.07	7.27–8.28	7.46–8.46
B-FeSMA-A	5	7.18	0.42	5.78	6.55–7.66	6.66–7.70
B-Steel-160	5	5.89	0.82	13.99	5.01–6.87	4.87–6.91
B-FeSMA-160	5	9.41	0.36	3.82	8.89–9.79	8.96–9.86

All strength quantities are in MPa. CIs are pointwise intervals for within-batch means.

**Table 8:** Thermal contrasts in peak strength calculated as heated minus ambient.

Comparison	Difference (MPa)	Change (%)	95% CI (MPa)	Adjusted *p*
B-HPC	−1.21	−28.0	−1.75 to −0.67	0.002652
B-Steel	−2.07	−26.0	−3.08 to −1.06	0.005145
B-FeSMA	+2.23	+31.1	1.66 to 2.80	0.000079
P-HPC	−1.40	−18.8	−2.33 to −0.47	0.009100

Five specimens per thermal group. Two-sided Welch tests; Holm-adjusted *p*-values across the four comparisons. Difference CIs are unadjusted 95% intervals, not simultaneous intervals.

**Table 9:** Approximate numerical peak strengths read from the vector curves in Figure 21, rounded to 0.1 MPa. All fibers are 20 mm long.

Sweep	Volume Fraction (%)	Diameter (mm)	Peak (MPa)
Content	1.0	0.5	7.5
Content	1.5	0.5	9.0
Content	2.0	0.5	9.6
Content	2.5	0.5	10.3
Content	3.0	0.5	10.8
Diameter	2.0	0.3	11.3
Diameter	2.0	0.4	10.8
Diameter	2.0	0.5	9.6
Diameter	2.0	0.6	9.2
Diameter	2.0	0.7	8.6

These are graphical estimates, not additional simulations or exact solver output. The 2.0%, 0.5 mm baseline appears in both sweeps. No combinations beyond the two listed sweeps were analyzed.

## Data Availability

The data supporting the findings of this study are available from the corresponding author upon reasonable request.

## Abbreviations

The following abbreviations are used in this manuscript:

CDP	Concrete damage plasticity
FE	Finite element
Fe-SMA	Iron-based shape memory alloy
FRC	Fiber-reinforced concrete
GUI	Graphical user interface
HPC	High-performance concrete
SMA	Shape memory alloy
SME	Shape memory effect
